# Magnesium Sulfate Mitigates the Progression of Monocrotaline Pulmonary Hypertension in Rats

**DOI:** 10.3390/ijms20184622

**Published:** 2019-09-18

**Authors:** Chao-Yuan Chang, Hung-Jen Shih, I-Tao Huang, Pei-Shan Tsai, Kung-Yen Chen, Chun-Jen Huang

**Affiliations:** 1 Department of Anesthesiology, Wan Fang Hospital, Taipei Medical University, Taipei 116, Taiwan; yuanc669@gmail.com; 2 Integrative Research Center for Critical Care, Wan Fang Hospital, Taipei Medical University, Taipei 116, Taiwan; jasta1206@gmail.com; 3 Graduate Institute of Clinical Medicine, College of Medicine, Taipei Medical University, Taipei 110, Taiwan; 4 Department of Urology, Wan Fang Hospital, Taipei Medical University, Taipei 116, Taiwan; 5 Department of Urology, School of Medicine, College of Medicine, Taipei Medical University, Taipei 110, Taiwan; 6 Medical Education Unit, Redcliffe Hospital, Metro North Hospital and Health Service, Queensland Government, Redcliffe, QLD 4020, Australia; 7 School of Public Health, Faculty of Medicine, University of Queensland, Herston, QLD 4006, Australia; 8 School of Nursing, College of Nursing, Taipei Medical University, Taipei 110, Taiwan; ptsai@tmu.edu.tw; 9 Department of Nursing, Wan Fang Hospital, Taipei Medical University, Taipei 116, Taiwan; 10 Research Center of Big Data and Meta-analysis, Wan Fang Hospital, Taipei Medical University, Taipei 116, Taiwan

**Keywords:** right heart failure, oxidation, mitochondria, inflammation, inflammasomes, apoptosis

## Abstract

We investigated whether magnesium sulfate (MgSO_4_) mitigated pulmonary hypertension progression in rats. Pulmonary hypertension was induced by a single intraperitoneal injection of monocrotaline (60 mg/kg). MgSO_4_ (100 mg/kg) was intraperitoneally administered daily for 3 weeks, from the seventh day after monocrotaline injection. Adult male rats were randomized into monocrotaline (MCT) or monocrotaline plus MgSO_4_ (MM) groups (*n* = 15 per group); control groups were maintained simultaneously. For analysis, surviving rats were euthanized on the 28^th^ day after receiving monocrotaline. The survival rate was higher in the MM group than in the MCT group (100% versus 73.3%, *p* = 0.043). Levels of pulmonary artery wall thickening, α-smooth muscle actin upregulation, right ventricular systolic pressure increase, and right ventricular hypertrophy were lower in the MM group than in the MCT group (all *p* < 0.05). Levels of lipid peroxidation, mitochondrial injury, inflammasomes and cytokine upregulation, and apoptosis in the lungs and right ventricle were lower in the MM group than in the MCT group (all *p* < 0.05). Notably, the mitigation effects of MgSO_4_ on pulmonary artery wall thickening and right ventricular hypertrophy were counteracted by exogenous calcium chloride. In conclusion, MgSO_4_ mitigates pulmonary hypertension progression, possibly by antagonizing calcium.

## 1. Introduction

Pulmonary hypertension, characterized by increased pulmonary artery resistance and poor compliance, may cause right ventricular hypertrophy, right heart failure, and even death [1,2]. Approved pulmonary hypertension medications (such as endothelial receptor antagonists and phosphodiesterase inhibitors) improve pulmonary functional capacity and cardiopulmonary hemodynamics [3,4]. However, these medications do not improve clinical outcomes [5]. A primary reason is that these medications cannot block the pathogenesis of pulmonary hypertension [3,4,5]. Pulmonary artery remodeling is crucial in mediating the development of pulmonary hypertension and right ventricular hypertrophy [6]. The triggering factors of pulmonary artery remodeling involve oxidation, mitochondrial injury, inflammation, and apoptosis [4,7,8,9]. Effective therapy for pulmonary hypertension is not currently available [10,11].

Magnesium sulfate (MgSO_4_) is a natural calcium antagonist [12]. MgSO_4_ is used clinically for the treatment of pre-eclampsia/eclampsia, arrhythmia, and asthma [13,14,15,16]. Notably, MgSO_4_ is a vasodilator [12]. MgSO_4_ also has potent anti-oxidant and anti-inflammation activities [12,16,17]. Moreover, MgSO_4_ can modulate mitochondrial dysfunction, inflammasomes, and apoptosis [18,19,20]. Therefore, we conjectured that MgSO_4_ may act by modulating pulmonary vascular resistance and the aforementioned mechanisms to exert beneficial effects against pulmonary hypertension.

Our conjecture was partially supported by previously reported clinical and laboratory data [21,22,23,24,25]. For example, short-term MgSO_4_ use (e.g., up to 36 h) has been shown to alleviate pulmonary vascular constriction in premature neonates and newborns with persistent pulmonary hypertension, and in experimental animals with pulmonary hypertension [21,22,23,24]. A laboratory study also demonstrated that pretreatment with magnesium aspartate for 2 weeks mitigated pulmonary hypertension in experimental rats [25]. However, these data have only demonstrated the beneficial effects of short-term MgSO_4_ therapy and the preventive effects of magnesium aspartate against pulmonary hypertension. The question of whether long-term MgSO_4_ therapy can exert beneficial effects against pulmonary hypertension remains unexplored. The effects of MgSO_4,_ administered after the induction of pulmonary hypertension, also remain unexplored.

Hence, we conducted the present study, which employed a widely used monocrotaline pulmonary hypertension rodent model [26,27]. We hypothesized that long-term MgSO_4_ therapy after the induction of pulmonary hypertension can mitigate pulmonary hypertension progression in monocrotaline-treated rats. The effects of long-term MgSO_4_ therapy on modulating oxidation, mitochondrial injury, inflammation, and apoptosis were also investigated.

## 2. Results

### 2.1. MgSO_4_ Improves Survival in Monocrotaline-Treated Rats

In the monocrotaline (MCT) group, four rats died within 28 days after monocrotaline treatment (on the 16^th^, 20^th^, 24^th^, and 28^th^ days). By contrast, all rats in the sham, MgSO_4_, and monocrotaline plus MgSO_4_ (MM) groups survived the experiment. Therefore, the 28-day survival rates in the sham, MgSO_4_, MCT, and MM groups were 100%, 100%, 73.3%, and 100%, respectively. Analysis revealed that the survival rate in the MM group was significantly higher than in the MCT group (*p* = 0.043). These results supported the therapeutic effects of long-term MgSO_4_ therapy against pulmonary hypertension in monocrotaline-treated rats.

### 2.2. MgSO_4_ Mitigates Monocrotaline-Induced Pulmonary Hypertension

**Figure 1:**
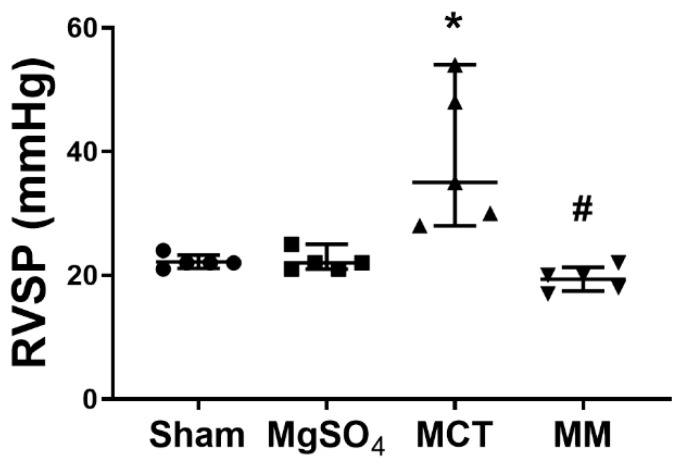
Magnesium sulfate (MgSO_4_) mitigates monocrotaline-induced increases in right ventricular systolic pressure (RVSP) in rats. MgOS_4_: MgSO_4_ (100 mg/kg/day) group. MCT: monocrotaline (60 mg/kg) group. MM: monocrotaline (60 mg/kg) plus MgSO_4_ (100 mg/kg/day) group. Data are expressed as median (range). RVSP data were obtained from five rats from each group. * *p* < 0.05 versus the sham group. # *p* < 0.05 versus the MCT group.

Pulmonary hypertension was evaluated in terms of right ventricular systolic pressure (RVSP). The mean RVSP in the sham group was 24 mmHg. We arbitrarily defined an RVSP of >30 mmHg to indicate the presence of pulmonary hypertension. Our data indicated that four out of five rats (80%) in the MCT group and no rats (0%) in the MM group had a RVSP of >30 mmHg. Statistical analysis revealed that the RVSPs in the sham and MgSO_4_ groups were low, whereas the RVSP in the MCT group was significantly higher than that in the sham group (*p* = 0.002; Figure 1). Notably, the RVSP in the MM group was significantly lower than that in the MCT group (*p* < 0.001; Figure 1). These findings revealed that long-term MgSO_4_ therapy effectively decreased pulmonary hypertension in monocrotaline-treated rats.

### 2.3. MgSO_4_ Mitigates Monocrotaline-Induced Pulmonary Artery Remodeling, Lung Tissue Lipid Peroxidation, and Pulmonary Artery Mitochondrial Injury

**Figure 2:**
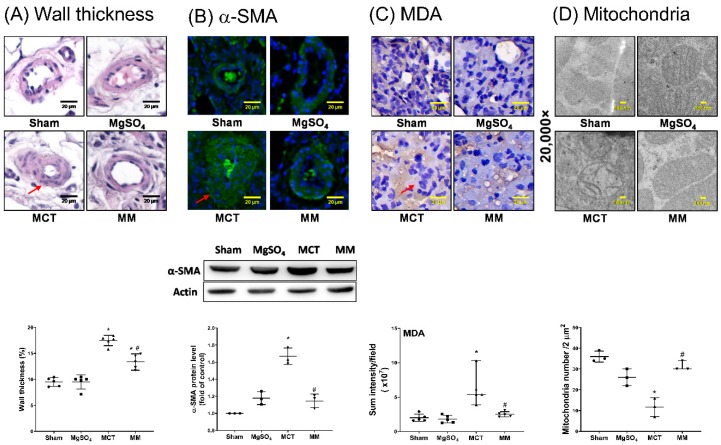
Magnesium sulfate (MgSO_4_) mitigates monocrotaline-induced pulmonary artery remodeling and lipid peroxidation in the lung tissues, and monocrotaline-induced mitochondrial injury in pulmonary arteries. (**A**) Representative microscopic images of the pulmonary arteries in the lung tissues stained with hematoxylin and eosin (200×). The red arrow indicates the representative image of thickened pulmonary arteries. Wall thickness ratios of pulmonary arteries are presented. (**B**) Representative microscopic images of α-smooth muscle actin (α-SMA, green fluorescence) and nuclei (blue fluorescence) of the pulmonary arteries in the lung tissues through immunofluorescence staining assay (200×). Representative gel photography of α-SMA and actin (internal standard) in the lung tissues were obtained through immunoblotting assays. The red arrow indicates the representative image of increased α-SMA expression. Quantitative sum intensities of α-SMA and relative α-SMA/actin band densities are presented. (**C**) Representative microscopic images of malondialdehyde (MDA, the end product of lipid peroxidation) in the lung tissues observed through immunohistochemistry staining assay (200×). The red arrow indicates a representative image of MDA with brown staining. Quantitative sum intensities of MDA are presented. (**D**) Representative transmission electron microscopic images of mitochondria in the pulmonary artery (20,000×). Mean intact mitochondria numbers (per 2 μm^2^) are presented. Sham: normal saline group. MgOS_4_: MgSO_4_ (100 mg/kg/day) group. MCT: monocrotaline (60 mg/kg) group. MM: monocrotaline (60 mg/kg) plus MgSO_4_ (100 mg/kg/day) group. Data are expressed as median (range). Data of the wall thickness ratio, α-SMA immunofluorescence staining, and α-SMA immunoblotting assays were obtained from five, four, and three rats from each group, respectively. Data of MDA and mitochondria were obtained from five and three rats from each group, respectively. * *p* < 0.05 versus the sham group. # *p* < 0.05 versus the MCT group.

Pulmonary artery remodeling was assayed by measuring wall thickness ratio and α-smooth muscle actin (α-SMA) expression in pulmonary arteries. Lipid peroxidation status was evaluated through immunohistochemistry assays for malondialdehyde (MDA) in the lung tissues to evaluate the pulmonary oxidative injuries induced by monocrotaline. The levels of mitochondrial injuries in the pulmonary arteries, measured by mean intact mitochondria number, were evaluated by transmission electron microscopy. Our data revealed that the wall thickness ratio for pulmonary arteries in the sham and MgSO_4_ groups were low, whereas the wall thickness ratio for pulmonary arteries in the MCT group was significantly higher than that in the sham group (*p* < 0.001; Figure 2A). In addition, the wall thickness ratio for pulmonary arteries in the MM group was significantly lower than that in the MCT group (*p* < 0.001; Figure 2A). Similar pictures between the groups were observed in the analyses of α-SMA expression (Figure 2B). These results demonstrated that long-term MgSO_4_ therapy effectively reduced monocrotaline-induced pulmonary artery remodeling. Similar pictures between the groups were also observed in the analyses of MDA’s expression in the lung tissue (Figure 2C), indicating that long-term MgSO_4_ therapy reduced monocrotaline-induced pulmonary oxidation. By contrast, the mean intact mitochondria numbers in the sham and MgSO_4_ groups were similar, whereas the mean intact mitochondria number in the MCT group was significantly lower than that in the sham group (*p* < 0.001; Figure 2D). Moreover, the mean intact mitochondria number in the MM group was significantly higher than that of the MCT group (*p* < 0.001; Figure 2D). These findings also revealed that long-term MgSO_4_ therapy reduced monocrotaline-induced mitochondria injury in the pulmonary artery.

### 2.4. MgSO_4_ Mitigates Monocrotaline-Induced Inflammation and Apoptosis in Lung Tissues

**Figure 3:**
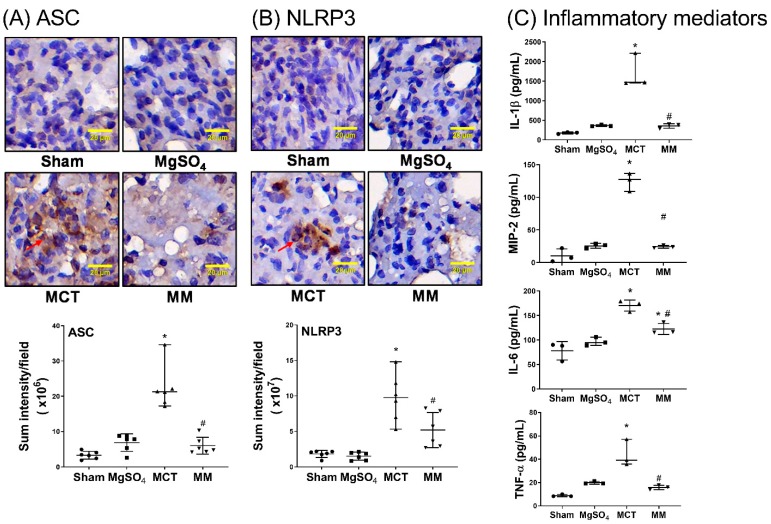
Magnesium sulfate (MgSO_4_) mitigates monocrotaline-induced upregulation of inflammasomes and inflammatory mediators in the lung tissues. Representative microscopic images of (**A**) apoptosis-associated speck-like protein containing a caspase recruitment domain (ASC, an adaptor protein for inflammasome receptors) and (**B**) nod-like receptor protein 3 (NLRP3, the crucial element of inflammasomes) in the lung tissues through immunohistochemistry staining assay (200×) are displayed. Quantitative sum intensities of ASC and NLRP3 are presented. Red arrows in (**A**) and (**B**) indicate the representative images of ASC and NLRP3 with brown color staining, respectively. (**C**) Pulmonary levels of interleukin-1β (IL-1β), IL-6, tumor necrosis factor-α (TNF-α), and macrophage inflammatory protein 2 (MIP-2) analyzed by enzyme-linked immunosorbent assay. Sham: normal saline group. MgOS_4_: MgSO_4_ (100 mg/kg/day) group. MCT: monocrotaline (60 mg/kg) group. MM: monocrotaline (60 mg/kg) plus MgSO_4_ (100 mg/kg/day) group. Data are expressed as median (range). Data of ASC, NLPR3, and inflammatory mediators were obtained from six, six, and three rats from each group, respectively. *p < 0.05 versus the sham group. # *p* < 0.05 versus the MCT group.

**Figure 4:**
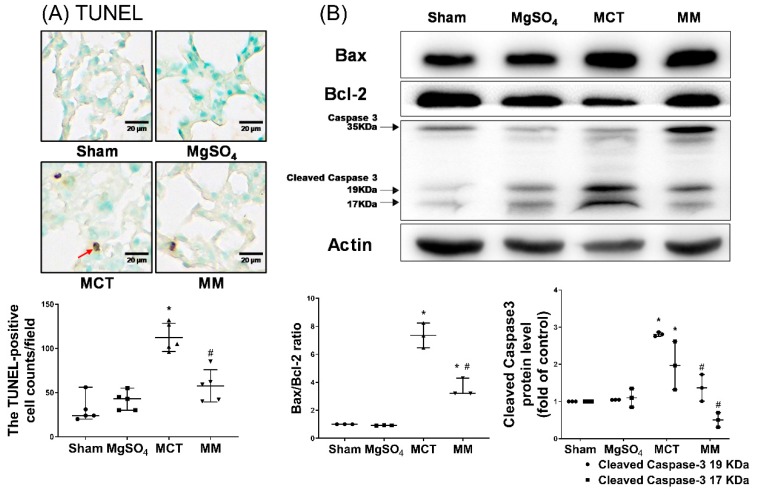
Magnesium sulfate (MgSO_4_) mitigates monocrotaline-induced apoptosis in the lung tissues. (**A**) Representative microscopic images of DNA fragmentation in the lung tissues as assayed by the terminal deoxynucleotidyl transferase dUTP nick end labeling (TUNEL) method (200×). The red arrow indicates the representative image of TUNEL-positive nuclei. Mean TUNEL-positive cell counts (per 0.25 mm^2^) are presented. (**B**) Representative gel photography of apoptosis-related proteins Bax, Bcl-2, caspase 3, and actin (internal standard) in the lung tissues obtained through immunoblotting assays. Bax/Bcl-2 ratios and relative cleaved caspase 3/actin band densities are presented. Sham: normal saline group. MgOS_4_: MgSO_4_ (100 mg/kg/day) group. MCT: monocrotaline (60 mg/kg) group. MM: monocrotaline (60 mg/kg) plus MgSO_4_ (100 mg/kg/day) group. Data are expressed as median (range). Data of TUNEL and the apoptosis-related protein were obtained from five and three rats from each group, respectively. * *p* < 0.05 versus the sham group. # *p* < 0.05 versus the MCT group.

Inflammatory status in the lung tissues was evaluated by the expression of inflammasome components, including apoptosis-associated speck-like protein containing a caspase recruitment domain (ASC) and nod-like receptor protein 3 (NLRP3), using immunohistochemistry staining assay; and inflammatory mediators, including interleukin-1β (IL-1β), IL-6, tumor necrosis factor-α (TNF-α), and macrophage inflammatory protein 2 (MIP-2), using enzyme-linked immunosorbent assay (ELISA). The pulmonary level of apoptosis was evaluated by terminal deoxynucleotidyl transferase dUTP nick end labeling (TUNEL) and immunoblotting assays for Bax, Bcl-2, and cleaved caspase-3. Our data revealed that the expression levels of ASC and NLRP3 in the lung tissues of the sham and MgSO_4_ groups were low, whereas those in the MCT group were significantly higher than those in the sham group (both *p* < 0.001, respectively; Figure 3A,B). Notably, the expression levels of ASC and NLRP3 in the lung tissues of the MM group were significantly lower than those in the MCT group (*p* < 0.001 and *p* = 0.007, respectively; Figure 3A,B). In addition, data of inflammatory mediators (i.e., IL-1β, IL-6, TNF-α, and MIP-2; Figure 3C), the TUNEL-positive cell counts (Figure 4A), Bax/Bcl-2 ratios, and expression levels of cleaved caspase-3 (Figure 4B) paralleled those of the ASC and NLPR3. These findings indicate that long-term MgSO_4_ therapy decreased monocrotaline-induced lung inflammation and apoptosis.

### 2.5. MgSO_4_ Mitigates Monocrotaline-Induced Right Ventricular Hypertrophy

**Figure 5:**
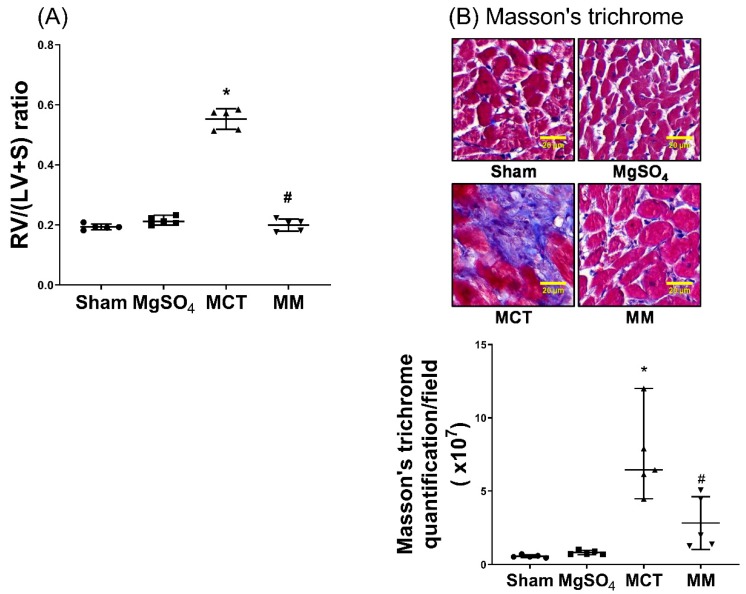
Magnesium sulfate (MgSO_4_) mitigates monocrotaline-induced right ventricular hypertrophy. (**A**) Weight ratios of right ventricle to left ventricle plus septum [RV/(LV+S)]. (**B**) Representative microscopic images of right ventricular fibrosis as assayed by Masson’s trichrome staining (200×). Quantitative sum intensities of collagen are presented. Blue, red, and dark blue staining indicates collagen, muscle fibers, and nuclei, respectively. Sham: normal saline group. MgOS_4_: MgSO_4_ (100 mg/kg/day) group. MCT: monocrotaline (60 mg/kg) group. MM: monocrotaline (60 mg/kg) plus MgSO_4_ (100 mg/kg/day) group. Data are expressed as median (range). Data of RV/(LV+S) and right ventricular fibrosis were both obtained from five rats from each group. * *p* < 0.05 versus the sham group. # *p* < 0.05 versus the MCT group.

Levels of right ventricular hypertrophy were assayed with respect to the right ventricle to left ventricle plus septum [RV/(LV+S)] ratio and fibrosis levels in the right ventricular myocardium using Masson’s trichrome staining assay. Our data revealed that the RV/(LV+S) ratio and right ventricular fibrosis levels in the sham and MgSO_4_ group were low, whereas those in the MCT group were significantly higher than those in the sham group (both *p* < 0.001; Figure 5A,B). In addition, the RV/(LV+S) ratio and right ventricular fibrosis level in the MM group were significantly lower than those in the MCT group (*p* < 0.001 and *p* = 0.012, respectively; Figure 5A,B). These findings indicate that long-term MgSO_4_ therapy decreased monocrotaline-induced right ventricular hypertrophy.

### 2.6. MgSO_4_ Mitigates Monocrotaline-Induced Lipid Peroxidation, Mitochondrial Injury, Inflammation, and Apoptosis in Myocardium

**Figure 6:**
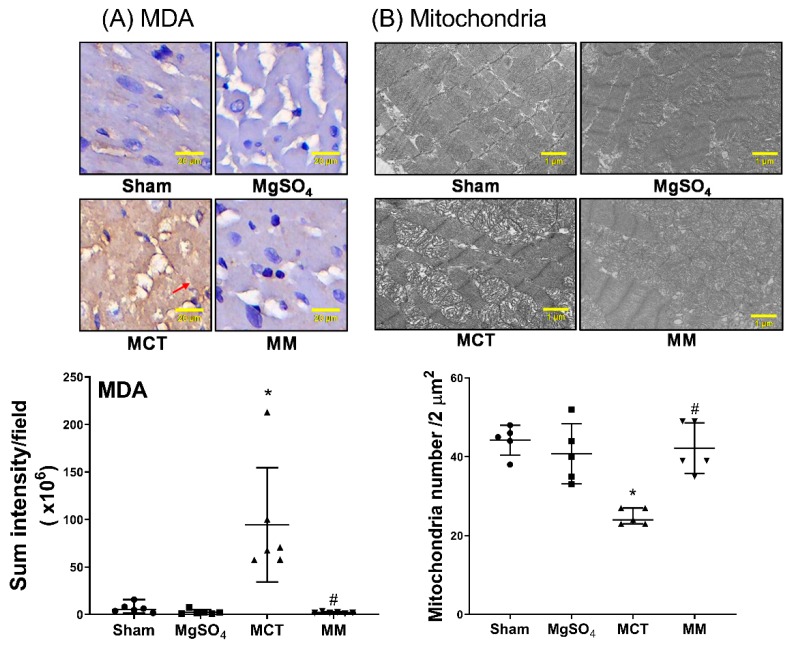
Magnesium sulfate (MgSO_4_) mitigates monocrotaline-induced lipid peroxidation and mitochondrial injury in the right ventricular myocardium. (**A**) Representative microscopic images of malondialdehyde (MDA, the end product of lipid peroxidation) in the right ventricular myocardium obtained using an immunohistochemistry staining assay (200×). The red arrow indicates the representative image of MDA with brown color staining. The quantitative sum intensities of MDA are presented. (**B**) Representative transmission electron microscopic images of mitochondria in the right ventricular myocardium (8000×). Mean intact mitochondria numbers (per 2 μm^2^) are presented. Sham: normal saline group. MgOS_4_: MgSO_4_ (100 mg/kg/day) group. MCT: monocrotaline (60 mg/kg) group. MM: monocrotaline (60 mg/kg) plus MgSO_4_ (100 mg/kg/day) group. Data are expressed as median (range). Data of MDA and mitochondria were obtained from six and five rats from each group, respectively. * *p* < 0.05 versus the sham group. # *p* < 0.05 versus the MCT group.

**Figure 7:**
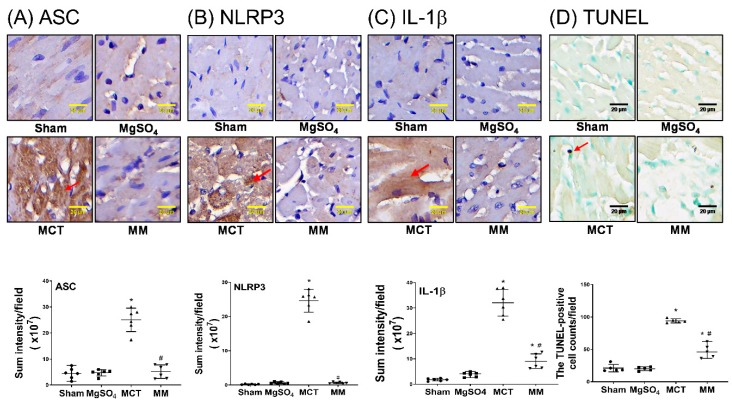
Magnesium sulfate (MgSO_4_) mitigates the monocrotaline-induced upregulation of apoptosis, inflammasomes, and the inflammatory mediator in the right ventricular myocardium. Representative microscopic images obtained from the immunohistochemistry staining assay of the right ventricular myocardium (200×). Quantitative sum intensities of (**A**) apoptosis-associated speck-like protein containing a caspase recruitment domain (ASC, an adaptor protein for inflammasome receptors), (**B**) nod-like receptor protein 3 (NLRP3, the crucial element of inflammasomes), and (**C**) interleukin-1β (IL-1β). Red arrows in (**A**), (**B**), and (**C**) indicate the representative images of ASC, NLPR3, and IL-1β, respectively. (**D**) Representative microscopic images of DNA fragmentation in the right ventricular myocardium, as assayed by the terminal deoxynucleotidyl transferase dUTP nick end labeling (TUNEL) method (200×). Mean TUNEL-positive cell counts (per 0.25 mm^2^) are presented. Red arrow indicates the representative images of TUNEL-positive nuclei. Sham: normal saline group. MgOS_4_: MgSO_4_ (100 mg/kg/day) group. MCT: monocrotaline (60 mg/kg) group. MM: monocrotaline (60 mg/kg) plus MgSO_4_ (100 mg/kg/day) group. Data are expressed as median (range). Data of ASC, NLRP3, IL-1β, and TUNEL were obtained from six, six, six, and five rats from each group, respectively. * *p* < 0.05 versus the sham group. # *p* < 0.05 versus the MCT group.

MDA levels were obtained using immunohistochemistry staining assay to evaluate the lipid peroxidation status of the right ventricular myocardium. The levels of mitochondrial injury, as measured by the mean intact mitochondria number, in the right ventricular myocardium, were assayed through transmission electron microscopy. The levels of inflammation, with respect to inflammasomes (ASC, NLRP3) and IL-1β, of the right ventricular myocardium, were obtained using immunohistochemistry staining assay. The TUNEL assay was also performed to evaluate the apoptosis status of the right ventricular myocardium. Our data revealed that the levels of MDA in the right ventricular myocardium of the sham and MgSO_4_ groups were low, whereas that of the MCT group was significantly higher than that of the sham group (*p* < 0.001; Figure 6A). In addition, the level of MDA in the right ventricular myocardium of the MM group was significantly lower than that of the MCT group (*p* < 0.001; Figure 6A). Data of mitochondrial injury (Figure 6B) and data of expression levels of ASC, NLRP3, IL-1β, and the TUNEL-positive cell counts in the right ventricle myocardium (Figure 7A–D), paralleled the data of MDA. These findings indicate that long-term MgSO_4_ therapy mitigated monocrotaline-induced oxidation, mitochondrial injury, inflammation, and apoptosis.

### 2.7. Calcium Chloride Counteracts the Effect of Mgso_4_ in Monocrotaline-Treated Rats

**Figure 8:**
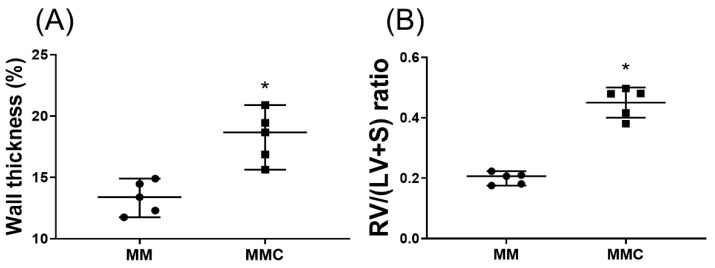
Calcium chloride counteracts the effects of magnesium sulfate (MgSO_4_) in monocrotaline-treated rats. (**A**) Wall thickness ratio of the pulmonary arteries. (**B**) Weight ratio of right ventricle to left ventricle plus septum [RV/(LV+S)]. MM: monocrotaline (60 mg/kg) plus MgSO_4_ (100 mg/kg/day) group. MMC: monocrotaline (60 mg/kg) plus MgSO_4_ (100 mg/kg/day) plus calcium chloride (100 mg/kg/day) group. Data are expressed as median (range). Data of wall thickness ratio of pulmonary arteries and RV/(LV+S) were both obtained from five rats from each group, respectively. * *p* < 0.05 the MMC group versus MM group.

MgSO_4_ is a natural calcium antagonist [12]. To determine whether MgSO_4_ acts by antagonizing calcium to exert its effects against monocrotaline pulmonary hypertension, another set of rats (*n* = 5; designated as the MMC group) were treated with monocrotaline. On the seventh day after monocrotaline, a daily intraperitoneal injection of 100 mg/kg of calcium chloride followed by 100 mg/kg of MgSO_4_ was administered over 3 weeks. On the 28^th^ day after monocrotaline administration, rats of the MMC group were euthanized. Wall thickness ratio of the pulmonary arteries and the RV/(LV+S) ratios were then measured. Our findings indicate that the wall thickness ratio of the pulmonary arteries and the RV/(LV+S) ratios in the MMC group were significantly higher than those in the MM group (both *p* < 0.001; Figure 8A,B). These findings indicate that exogenous calcium counteracted the effects of long-term MgSO_4_ therapy, reducing pulmonary artery wall thickening and right ventricular hypertrophy induced by monocrotaline.

## 3. Discussion

This study is the first to demonstrate that long-term MgSO_4_ therapy, upon the induction of pulmonary hypertension, improves survival in monocrotaline-treated rats. This study also demonstrated that long-term MgSO_4_ therapy, upon the induction of pulmonary hypertension, mitigates the development of monocrotaline-induced pulmonary hypertension and right ventricular hypertrophy. Collectively, our findings confirmed our hypothesis and provided clear evidence of the therapeutic effects of long-term MgSO_4_ therapy in counteracting monocrotaline pulmonary hypertension progression. These findings improve our understanding of the therapeutic potential of MgSO_4_. These findings also support the strategy of incorporating MgSO_4_ into pulmonary hypertension therapy. Because MgSO_4_ is inexpensive and already in clinical use, this study has crucial clinical implications. However, because this study is a rodent study, our findings should be extrapolated with caution; additional studies on the translational use of MgSO_4_ in patients with pulmonary hypertension is required.

Studies have reported the crucial roles of oxidation, mitochondrial injury, inflammation, and apoptosis in triggering the development of pulmonary artery remodeling and right ventricular hypertrophy in pulmonary hypertension [4,9,10,11]. This finding is corroborated by our study because our data demonstrated significant lipid peroxidation, mitochondrial injury, inflammation, and apoptosis in the lung tissues and right ventricular myocardium in monocrotaline-treated rats. Notably, previous findings from studies conducted by our own and other research groups have demonstrated the effects of MgSO_4_ on modulating oxidation, mitochondrial dysfunction, inflammation, and apoptosis [12,16,17,18,19,20]. From these previous findings, we conjectured that MgSO_4_ exerts significant effects in the modulation of these crucial mechanisms. Our current findings support this conjecture. Furthermore, our study is the first to demonstrate that long-term MgSO_4_ therapy can mitigate monocrotaline-induced lipid peroxidation, mitochondrial injury, inflammation, and apoptosis in lung tissues and the right ventricular myocardium. Thus, we inferred that long-term MgSO_4_ therapy can mitigate monocrotaline-induced pulmonary artery remodeling, pulmonary hypertension, and right ventricular hypertrophy.

**Figure 9:**
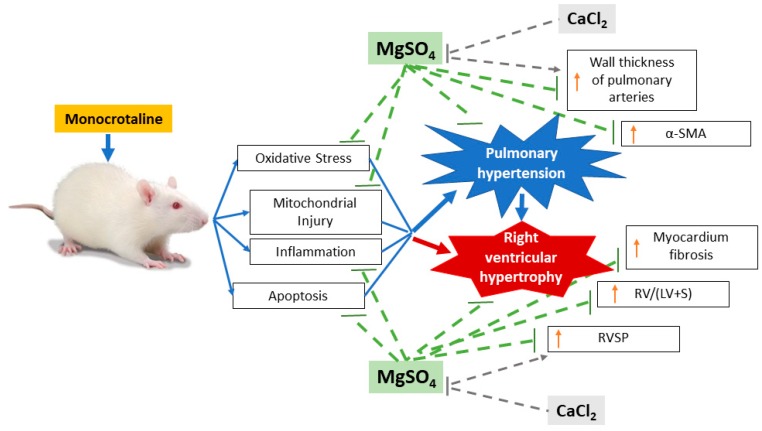
Illustration of the effects of magnesium sulfate (MgSO_4_) on inhibiting monocrotaline-induced pulmonary hypertension and right ventricular hypertrophy in rats. Counteracting effects of calcium chloride (CaCl_2_) on the therapeutic effects of MgSO_4_ are also illustrated. α-SMA: α-smooth msucle actin. RV/(LV+S): the weight ratio of right ventricle to left ventricle plus septum. RVSP: right ventricular systolic pressure.

Although data from this study provide clear evidence for the effects of long-term MgSO_4_ therapy in counteracting pulmonary hypertension, the mechanisms underlying the effects of MgSO_4_ remain unclear. Studies on the aforementioned mechanisms have indicated that a rise in intracellular calcium level is a major factor involved in triggering pulmonary vasoconstriction, pulmonary artery smooth muscle cell proliferation, and subsequent pulmonary artery remodeling [28]. In addition, a rise in intracellular calcium levels induced by reactive oxygen species mediates mitochondrial injury and apoptosis in pulmonary artery smooth muscle cells and right ventricular myocytes in pulmonary hypertension [29,30,31]. Because MgSO_4_ is a natural calcium antagonist [12], we conjectured that MgSO_4_ antagonizes calcium and alleviated pulmonary hypertension. A similar mechanism has been proposed by a previous magnesium aspartate study [25]. This mechanism of calcium antagonism is indicated by this study’s findings; our data revealed that exogenous calcium partially counteracted the therapeutic effects of MgSO_4_ in terms of the mitigation of monocrotaline-induced pulmonary artery wall thickening and right ventricular hypertrophy. The possible mechanisms and aforementioned effects of long-term MgSO_4_ therapy against pulmonary hypertension are summarized in Figure 9.

Therefore, medications that can antagonize calcium may exert effects similar to those of MgSO_4_ in pulmonary hypertension therapy. This similarity in their effects is supported by clinical data that calcium channel blockers (e.g., nifedipine, diltiazem, and amlodipine) may mitigate pulmonary hypertension, particularly in patients with pulmonary hypertension with an acute response to vasodilator testing [32]. However, the clinical use of calcium channel blockers against pulmonary hypertension cannot be recommended due to the current lack of solid evidence [33]. The question of whether MgSO_4_ can offer better therapeutic effects relative to calcium channel blockers remains unanswered. Nevertheless, our findings that long-term MgSO_4_ therapy can mitigate oxidation, mitochondrial injury, inflammation, and apoptosis, thus exerting beneficial effects against pulmonary hypertension, provide partial support for the clinical use of MgSO_4_ against pulmonary hypertension.

MgSO_4_ is regularly used in clinical practice. The established indications of MgSO_4_ include pre-eclampsia/eclampsia, arrhythmia, and asthma [12,13,14,15]. Although the present study and previous clinical and laboratory studies [21,22,23,24] have indicated that MgSO_4_ may exert certain beneficial effects against pulmonary hypertension, evidence that conclusively supports the clinical use of MgSO_4_ against pulmonary hypertension is not currently available [34]. A primary concern is that MgSO_4_ is not a selective pulmonary vasodilator [12]. Theoretically, MgSO_4_ may also cause systemic hypotension, which in turn increases ventilation perfusion mismatching and worsens hypoxia in pulmonary hypertension. Because this study did not measure systemic blood pressure and arterial blood gas, these adverse effects of MgSO_4_ remain to be elucidated. However, our data revealed that all rats receiving monocrotaline plus MgSO_4_ survived the experiment. A previous clinical study of premature neonates with persistent pulmonary hypertension also revealed that short-term MgSO_4_ therapy did not cause significant side effects [21]. Images similar to ours were obtained in a previous clinical study of newborns with severe persistent pulmonary hypertension—with the exception of one subject (out of 28 subjects), who developed systemic hypotension upon short-term MgSO_4_ therapy [22]. These data seem to indicate that short-term and long-term MgSO_4_ therapies were well tolerated in cases of pulmonary hypertension. However, a previous laboratory study reported that in lambs, MgSO_4_ potentially caused significant decreases in systemic vascular resistance, stoke volume index, and systemic blood pressure in hypoxia-induced pulmonary hypertension [24]. More studies are required before further conclusions can be drawn.

Because the definitive molecular etiology of human pulmonary hypertension remains unknown, the optimal animal model of pulmonary hypertension is also unknown [35]. Nevertheless, several mechanistic origins of pulmonary hypertension have been proposed [35]. Because inflammation is identified as a crucial mechanistic origin of pulmonary hypertension, inflammation-related models (i.e., monocrotaline) have been developed [35]. A single injection of monocrotaline is well-established to potentially cause inflammation and readily induce pulmonary hypertension development in animals, with the characteristics of pulmonary vascular remodeling, smooth muscle cell proliferation, endothelial dysfunction, and right ventricle failure [36]. Because these characteristics mimic human pulmonary hypertension, this animal model is widely used in studies of experimental pulmonary hypertension [25,26,27,36]. Thus, we believe that this model is suitable for the study of inflammation modulation–related pulmonary hypertension therapy. Because the therapeutic potential of MgSO_4_ depends largely on its anti-inflammation effects, this study chose to employ the monocrotaline model.

Notably, the increase in vascular tone has also been proposed as a crucial mechanistic origin of pulmonary hypertension [35,37]. Therefore, tone-related models (e.g., hypoxia with or without Sugen) have been developed and widely employed in studies of experimental pulmonary hypertension [35,37]. Because this study did not employ the hypoxia/Sugen pulmonary hypertension model, the question of whether long-term MgSO_4_ therapy can exert significant effects against hypoxia/Sugen-induced pulmonary hypertension remains unanswered.

Our current findings indicate the therapeutic effects of long-term MgSO_4_ therapy against monocrotaline-induced pulmonary hypertension in rats. Oxidation and mitochondrial dysfunction have been discovered to play crucial roles in mediating hypoxia/Sugen-induced pulmonary hypertension development in animals [37]. Because data from this study demonstrated the effects of long-term MgSO_4_ therapy in mitigating oxidation and mitochondrial dysfunction in monocrotaline-treated rats, we infer that long-term MgSO_4_ therapy potentially counteracts hypoxia/Sugen-induced pulmonary hypertension. This inference is partially supported by a previous laboratory study that demonstrated the potential of MgSO_4_ to mitigate hypoxia-induced pulmonary hypertension [24]. However, evidence for MgSO_4_′s therapeutic effects in hypoxia/Sugen-induced pulmonary hypertension is inconclusive and further study is required.

Notably, this study was designed to be a proof of concept. Therefore, only one dosage of MgSO_4_ was tested in this this study. The lowest effective dosage of MgSO_4_ is currently unknown. Questions of whether the therapeutic effects of MgSO_4_ are dose-dependent, and whether the prolonged use of MgSO_4_ causes toxicity also remain unanswered. Because information on the dosage range of long-term MgSO_4_ therapy and administration routes was scarce, we developed the current regimen of a daily intraperitoneal injection of 100 mg/kg MgSO_4_, modified from our previous sepsis study data [23]. Because we did not evaluate the resulting plasma levels of MgSO_4_, the associated kinetics of this regimen remain unstudied. In addition, oral administration, rather than intraperitoneal injection, is arguably easier to transpose to potential clinical use in patients. However, certain maneuvers (e.g., orogastric gavage) are required for effective oral administration in rats. Because this maneuver might cause stress in rats, we selected intraperitoneal injection for MgSO_4_ administration in this study. Crucially, this study provided evidence for the concept and underscored the therapeutic potential of long-term MgSO_4_ therapy against pulmonary hypertension. Future studies should investigate safety and effectiveness with regard to dosage range, administration routes, and associated kinetics to facilitate the development of more effective therapeutic regimens of MgSO_4_ against pulmonary hypertension.

Our study has the following limitations. First, this study investigated the possible role of calcium antagonism in mediating the therapeutic potential of MgSO_4_ against pulmonary hypertension, in addition to its primary aim. To reduce animal use in this study, we investigated only two parameters (i.e., wall thickness ratio of the pulmonary arteries and right ventricular hypertrophy) for experiments on calcium antagonism. However, because only two parameters were investigated, our findings provide only partial evidence for the mediating role of calcium antagonism. Future studies on multiple parameters, and more numerous controls of calcium and magnesium (e.g., calcium sulfate and magnesium chloride), may provide more robust evidence than the current study. Second, this study did not measure intracellular calcium levels. This study also did not investigate the effects of MgSO_4_ in terms of modulating the regulatory pathways of intracellular calcium signaling (e.g., voltage-gated calcium channels and non-voltage-dependent store-operated calcium entry) [38]. Moreover, potassium, sodium, and chloride ions are also involved in pulmonary hypertension development [38]. The question of whether these ions and their regulatory pathways also mediate the therapeutic effects of long-term MgSO_4_ therapy against pulmonary hypertension remains unanswered. Third, this study demonstrated that the monocrotaline-induced upregulation of inflammasomes (ASC and NLRP3) in lung tissues and MgSO_4_ could mitigate these effects, but the specific pulmonary cells expressing ASC and NLRP3 were not characterized. Fourth, this study did not compare therapeutic effects between MgSO_4_ and the vasoactive drugs already in clinical use in pulmonary hypertension patients (e.g., phosphodiesterase inhibitors and prostacyclin). Therefore, the question of whether MgSO_4_ offers better therapeutic effects than existing therapies against pulmonary hypertension remains unanswered. Fifth, monocrotaline can also cause liver and kidney injuries [39]. Because we did not investigate the effects of MgSO_4_ on mitigating liver and kidney injuries in monocrotaline-treated rats, such effects of long-term MgSO_4_ therapy remain to be elucidated. Sixth, this study did not investigate catecholamine levels. Pulmonary hypertension is associated with the activation of the sympathetic nervous system, and increased catecholamine levels are involved in pulmonary vascular remodeling [40]. Notably, MgSO_4_ can inhibit catecholamines [41], thus MgSO_4_ may also likely inhibit catecholamines while exerting its effects against pulmonary hypertension in monocrotaline-treated rats. However, because this study did not investigate catecholamine levels, it could not demonstrate such an inhibition of catecholamines. Seventh, recent advances in pathophysiology and cellular mechanisms have highlighted novel mechanisms and potential therapeutic targets of pulmonary hypertension. For example, endothelium-to-mesenchymal transition, high glucose levels, insulin resistance, disturbed blood flow, and oxidative stress in the induction of endothelial dysfunction have been discovered to be crucial mechanisms in mediating the development of pulmonary hypertension [42]. Future studies are required to elucidate the effects of MgSO_4_ on these crucial mechanisms. Finally, our findings do not conclusively warrant the use of MgSO_4_ in pulmonary hypertension therapy. Nonetheless, this study is a successful proof of concept that warrants future preclinical and translational research for investigating its clinical applicability. Crucially, these future studies should accord with the rigorous methodology of Provencher et al. [43]. Studies should use more than one animal model, different strains of animals, subjects of both sexes, a large sample size, the randomization and allocation concealment of animal intervention, and the blinding of outcome assessment, in addition to the proper analysis and interpretation of transparent data.

In conclusion, long-term MgSO_4_ therapy mitigates monocrotaline-induced pulmonary hypertension progression. Long-term MgSO_4_ therapy also mitigates monocrotaline-induced oxidation, mitochondrial injury, inflammation, and apoptosis. Antagonizing calcium is a potential mechanism.

## 4. Materials and Methods

### 4.1. Animals

Adult male Sprague–Dawley rats (200–250 g; BioLASCO, Taipei, Taiwan) were used for our experiments. The animal study was approved by the Institutional Animal Care and Use Committee, Taipei Medical University (LAC-2017-0288, 19-09-2017). The care and handling of the rats complied with the guidelines of the US National Institutes of Health.

### 4.2. Monocrotaline Pulmonary Hypertension Model and Dosages of Monocrotaline and MgSO_4_

In this study, the monocrotaline model was used, where a single injection of monocrotaline would induce pulmonary hypertension, within 3 to 4 weeks, in rats [26,27]. To minimize the number of animals used in this study, we performed a preliminary study to determine the lowest effective dosage of monocrotaline that induces pulmonary hypertension. Preliminary data revealed that in rats receiving 60 and 120 mg/kg of monocrotaline (Sigma-Aldrich, St. Louis, MO, USA), the RV/(LV+S) ratios were comparable, and both were significantly higher than those in rats receiving normal saline (*p* = 0.002 and 0.029, respectively). These data indicated that 60 and 120 mg/kg of monocrotaline exerted similar effects on inducing right ventricular hypertrophy. Thus, we chose to use 60 mg/kg of monocrotaline in this study. In addition, in this study, we administered a daily injection of 100 mg/kg MgSO_4_ (Sigma-Aldrich, St. Louis, MO, USA), as our previous data revealed that 100 mg/kg MgSO_4_ attenuated the adverse effects of sepsis [44].

### 4.3. Study Protocols

The rats were randomized into two groups (*n* = 15 for each group). One group received an intraperitoneal injection of monocrotaline (60 mg/kg) and another received monocrotaline (60 mg/kg) plus MgSO_4_ (100 mg/kg/day). These were designated the MCT and MM groups, respectively. Another set of rats was randomized into two groups; one received an intraperitoneal injection of normal saline (the sham group), and the other received injections of normal saline plus MgSO_4_ (100 mg/kg/day) (the MgSO_4_ group). These two groups served as the controls of vehicle and MgSO_4_, respectively (*n* = 15 in each group). On the first day of the study, monocrotaline and normal saline were administered to the MCT and sham group, respectively. MgSO_4_ was administered daily for 3 weeks, beginning from the seventh day after monocrotaline and normal saline injection in the MM and the MgSO_4_ groups, respectively. All rats were closely monitored for 28 days.

On the 28th day after the administration of monocrotaline and normal saline, the surviving rats were anesthetized with an intraperitoneal injection of ketamine/xylazine (60/8 mg/kg). Tracheostomy was performed; a tracheostomy tube (22-gauge angiocatheter) was inserted, and all rats were ventilated using a small animal ventilator (Harvard MiniVent; tidal volume: 325 µL; rate: 150 stroke/min; Harvard Apparatus, South Natick, MA, USA).

### 4.4. RVSP and RV/(LV+S) Ratio Measurements

Sternotomy was performed. A 25-gauge needle, connected to a pressure transducer (BIOPAC System, Model MP100, Santa Barbara, CA, USA), was inserted into the right ventricle to measure RVSP, as described in a previous study [45]. After RVSP measurement, the rats were euthanized by decapitation, and the heart was harvested. Atria were removed from the heart and the right ventricle was then dissected from the left ventricle and septum. The samples were weighed and RV/(LV+S) ratios were calculated.

### 4.5. Assay of Wall Thickness of Pulmonary Arteries

The lungs were prepared as previously described [44]. For pulmonary arteries corresponding to respiratory and terminal bronchioles, medial wall thicknesses and the external diameters were measured. Subsequently, the wall thickness ratio of each pulmonary artery was calculated using the formula 2 × medial wall thickness/external diameter [46].

### 4.6. Immunofluorescence Staining

Assay of the pulmonary artery smooth muscle cell proliferation was performed using immunofluorescence staining of α-SMA in pulmonary arteries, as described in a previous study [47]. Lung tissue sections were blocked, followed by incubation with a monoclonal mouse anti-SMA antibody (4 °C overnight; Santa Cruz, Dallas, TX, USA). Subsequently, the sections were incubated with a fluorescein isothiocyanate-conjugated donkey anti-goat-IgG secondary antibody (1:1000; Thermo Fischer, Waltham, MA, USA) and mounted with a mounting medium containing 4,6-diamidino-2-phenylindole (DAPI) (Thermo Fischer, USA). All sections were scanned (TissueFAXS; TissueGnostics Gmbh, Vienna, Austria) and analyzed (Image J; software available at https://imagej.nih.gov/ij/).

### 4.7. Immunoblotting Assay

Immunoblotting assays were performed for detecting α-SMA and apoptosis-related proteins in the lung tissues. The lung tissues were homogenized and centrifuged, and the supernatants were collected for analysis, as described in a previous study [44]. Equal amounts of proteins (65 μg) were separated through electrophoresis and transferred onto nitrocellulose membranes (Bio-Rad Laboratories, Hercules, CA, USA). The membranes were incubated overnight with the primary antibody of α-SMA (Sigma-Aldrich), apoptosis-related proteins (including Bax, Bcl-2, and caspase 3; all from Abcam, Cambridge, UK), or actin (as the internal standard; Sigma-Aldrich, St. Louis, MO, USA). Bound antibodies were detected by chemiluminescence (ECL plus kit; Amersham BioSciences, Buckinghamshire, UK) and analyzed (Image J). The ratio of Bax to Bcl-2 (i.e., the pro versus anti-apoptosis proteins) was calculated.

### 4.8. Enzyme-linked Immunosorbent Assay

The expression of inflammatory molecules in the lung tissues was obtained using enzyme-linked immunosorbent assay (ELISA, R & D Systems, Minneapolis, USA). Lung tissues were processed as per a previous study [44], and the levels of inflammatory molecules in the lungs, such as those of IL-1β, IL-6, TNF-α, and MIP-2, were measured using commercial kits (Enzo Life Science, Farmingdale, NY, USA).

### 4.9. Terminal Deoxynucleotidyl Transferase dUTP Nick End Labeling

DNA fragmentation (the key characteristic of apoptosis) in the lung tissues and right ventricular myocardium were evaluated using the transferase dUTP nick end labeling (TUNEL) method with an *in situ* cell death detection kit (Roche, Indianapolis, IN, USA), as previously described [48]. The staining of the apoptotic cells in the lung tissues and right ventricular myocardium was performed according to the manufacturer’s protocols. The tissues were also stained with DAPI (Thermo Fisher, Waltham, MA, USA) to detect total nuclei. All sections were scanned (TissueFAXS, Vienna, Austria). Five random fields (0.25 mm^2^) were selected for TUNEL-positive cell counting.

### 4.10. Immunohistochemistry Staining Assay

Immunohistochemistry staining assays on the paraffin sections of the lung tissues and right ventricular myocardium were performed to evaluate lipid peroxidation and inflammasomes, as described in a previous study [49]. Tissue sections were incubated with the primary antibody of MDA (the end product of lipid peroxidation), ASC (an adaptor protein for inflammasome receptors), or NLRP3 (the crucial element of inflammasomes) (all purchased from R&D Systems, Minneapolis, MN, USA). In addition, right ventricular myocardium tissue sections were incubated with an IL-1β primary antibody (R&D Systems, Minneapolis, MN, USA) to evaluate inflammatory mediator expression. All sections were incubated with horseradish peroxidase-conjugated secondary antibodies (EnVision System, Santa Clara, CA, USA), stained with 3,3′-Diaminobenzidine substrate chromogen and Mayer’s hematoxylin solutions (both from Thermo Fisher, Waltham, MA, USA), and followed by scanning (TissueFAXS, Vienna, Austria) and analysis (Image J).

### 4.11. Masson’s Trichrome Staining

Masson’s trichrome staining was performed on paraffin sections of the right ventricular myocardium to evaluate right ventricular fibrosis, as described in a previous study [50]. A commercial trichrome staining kit (Abcam, Cambridge, UK) was used for staining. Blue, red, and dark blue staining indicated collagen, muscle fibers, and nuclei, respectively. All sections were scanned (TissueFAXS, Vienna, Austria) and analyzed (Image J).

### 4.12. Transmission Electron Microscopic Analysis

The main pulmonary artery and right ventricular myocardium tissues were fixed, as per the method of a previous study [51]. The sections were examined under a transmission electron microscope (Hitachi HT-7700; Hitachi, Ltd., Tokyo, Japan). Mitochondrial injury was defined as either a deformation of the mitochondrial cristae or a disruption of the inner mitochondrial membrane, as discussed in a previous study [51]. Five random fields (2 μm^2^) were selected for counting the number of intact mitochondria.

### 4.13. Statistical Analysis

Data were expressed as medians (range). Between-group differences were analyzed using one-way analysis of variance (ANOVA) and post hoc pairwise comparisons with Tukey’s test. Kaplan–Meier survival analysis was preformed to determine between-group differences in the 28-day survival rates. Between-group differences in vascular responsiveness were analyzed using two-way ANOVA and Tukey’s test. A *p* value <0.05 was considered significant. A commercial package (SPSS 21.0, SPSS Inc., IBM Corporation, Somers, NY, USA) was used for statistical analyses.
